# Preventive spare parts supply strategy for large-scale equipment based on performance degradation

**DOI:** 10.1371/journal.pone.0348476

**Published:** 2026-06-17

**Authors:** Yongjia Gao, Jieyu Liu, Can Li, Qiang Shen, Ruiyan Zhang, Zhichao Feng

**Affiliations:** 1 The Rocket Force University of Engineering, Xi’an, Shaanxi, P.R. China; 2 The University of Science and Technology Beijing, Beijing, Beijing, P.R. China; Beijing Institute of Technology, CHINA

## Abstract

Timely replacement of spare parts can improve the reliability of large-scale equipment operation and is the critical link of preventive maintenance. This paper studies how to reasonably formulate spare parts supply strategy in large-scale equipment maintenance. Given the shortcomings of the conventional fixed cycle replacement method of equipment spare parts, a spare parts replacement method based on large-scale equipment performance degradation is proposed. The proposed method can shorten downtime and avoid waste. Firstly, by analyzing the large-scale equipment’s performance state characteristics, the performance evaluation model of the equipment is established using the interval evidential reasoning approach, and the envelope of performance degradation can be obtained. Weibull distribution is used to fit the performance degradation envelope, and the bisection method and maximum likelihood estimation method are combined to identify the model parameters. The health degree envelope of large-scale equipment performance is obtained. Then, the number of spare parts required is calculated based on health degree expectation. Considering the time required for ordering and transporting spare parts, the spare parts ordering strategy is formulated under the condition of large-scale equipment working simultaneously. Finally, the proposed method is applied to a type of large-scale equipment to verify the effectiveness of the proposed method.

## 1. Introduction

large-scale equipment-intensive industries such as steel manufacturing, automotive, and aerospace, equipment experiences continuous performance degradation due to the combined effects of internal and external factors. Failure to perform timely maintenance can lead to safety hazards and economic losses [1,2]. To ensure stable equipment operation, critical components that are structurally complex, difficult to manufacture, and have long production lead times need to be pre-stocked as spares [3,4]. Holding too few spares makes it difficult to meet maintenance demands, while holding too many results in resource waste [5,6]. Therefore, how to rationally allocate and utilize equipment support resources to maximize the overall operational efficiency of equipment has become an urgent problem to be addressed in spare parts management [7,8].

In engineering practice, there exists a class of equipment for which, once performance degradation or failure occurs, maintenance is extremely difficult and costly, and the prolonged downtime caused by maintenance results in substantial losses [9,10]. Such equipment is referred to as non-repairable [11], and replacement is the only feasible restoration action [12]. For non-repairable equipment, commonly used spare parts management strategies based on time-based maintenance (TBM) include age replacement [13,14], block replacement [15,16], repair-limit strategies [17,18], and fault-limit strategies [19,20]. These strategies rely on statistical analysis of historical failure data to determine replacement intervals [21], basing replacement decisions entirely on statistical regularities of historical data while ignoring the actual health condition of the equipment during operation [22]. The direct consequences are as follows: when equipment performance has deteriorated to the failure threshold but the scheduled replacement time has not yet been reached, delayed replacement leads to prolonged downtime [23]; conversely, when the equipment is still in good condition but the predetermined replacement interval has been reached, premature replacement results in unnecessary waste. In recent years, significant progress has been made in the joint optimization of maintenance and spare parts inventory. Yang et al. proposed a dynamic condition-based maintenance strategy based on an improved reinforcement learning framework for redundant systems, enabling flexible selection of imperfect maintenance or replacement actions according to the system state [24]. Wang et al. developed a joint optimization model of maintenance strategy and two-way inventory transshipment for balanced systems, using a semi-Markov decision process that simultaneously considers vertical and horizontal transshipment strategies [25]. Wang et al. also established an integrated optimization model of quality control and maintenance strategies for production systems, utilizing renewal process theory to address quality-related failures [26]. These methods belong to condition-based maintenance (CBM), whose core principle is to dynamically decide whether to perform replacement based on the current or recently observed system state. Compared with traditional TBM strategies, CBM can respond in real time to the actual health condition of equipment, avoiding unnecessary premature or delayed replacements, thereby reducing maintenance costs, minimizing unplanned downtime, and improving spare parts utilization efficiency [27].

However, the above condition-based maintenance methods mainly make decisions based on the current or recently observed system state, making it difficult to predict the remaining useful life of equipment in advance. If the remaining useful life of non-repairable equipment could be predicted in advance, spare parts ordering and transportation could be arranged more efficiently, thereby avoiding prolonged downtime caused by stockouts. In fact, equipment performance degradation is a gradual process that exhibits detectable early signs [28], which makes performance prediction possible. With the development of sensor technology, online monitoring of equipment and analysis of the trends of its performance indicators have enabled real-time assessment of the actual health condition of equipment [29,30].

For non-repairable large-scale equipment with small samples and complex indicators, machine learning-based methods (e.g., neural networks [31], support vector machines [32]) are highly prone to overfitting due to the scarcity of data; expert knowledge-based methods (e.g., fuzzy comprehensive evaluation [33], analytic hierarchy process [34]) rely heavily on human experience and cannot objectively and quantitatively capture the subtle degradation characteristics of complex large-scale equipment. The interval evidence reasoning (IER) method, developed from evidence theory, can effectively fuse qualitative knowledge and quantitative information to handle uncertainty in the evaluation process [35–39]. It has low dependence on equipment mechanism information and accurate physical models, making it more suitable for performance assessment under small-sample conditions [40]. However, the evaluation results output by the IER method often vary with the evaluated object, lacking a unified quantitative scale [41]. To address this issue, a “health degree” indicator needs to be introduced to uniformly measure the performance degradation of complex systems. The health degree of equipment refers to the quantitative measure of the state or capacity of a system to achieve its intended functions [42]. To adopt a unified and reliable indicator for measuring equipment performance degradation, a relationship between product performance and health degree should be established. That is, the laws that affect reliability and availability should be identified from the trend of equipment performance degradation, and preventive replacement should be triggered when the health degree reaches a certain threshold, based on which maintenance strategies are formulated [43,44]. Therefore, establishing a mapping between performance and health degree is the key to enabling health degree to serve as the basis for spare parts management.

Following the above reasoning, this paper first uses the interval evidence reasoning method to obtain the performance state of large-scale equipment, then maps the evaluation results to a health degree indicator, and finally fits the performance degradation envelope using a three-parameter Weibull distribution to obtain a probabilistic degradation function of the equipment health degree. This enables the prediction of when the equipment will reach the replacement threshold in the future, allowing spare parts ordering to be completed with consideration of the transportation lead time.

The rest of this paper is structured as follows. In Section 2, the problems needed to be studied are formulated. The performance envelope of the same batch of equipment is obtained with the IER approach in Section 3. The Weibull distribution is used to fit the large-scale equipment performance degradation envelope, and the large-scale equipment’s health degree curve is obtained in Section 4. Then, based on the health degree, the replacement strategy of spare parts for large-scale equipment is proposed in Section 5. A case study is introduced in Section 6 to verify the efficient of the proposed method. The conclusion is obtained in Section 7.

## 2. Problem formulation

This paper considers large-scale equipment performance as the basis of the spare parts replacement strategy. The relationship between large-scale equipment performance and health degree determines the replacement moment and the number of spare parts. The proposed method decomposes the preventive spare parts supply decision into three sequential and coupled problems. The output of each problem provides the input for the next one.

**Problem 1:** The large-scale equipment’s performance can reflect the working state’s degradation level. Accurate performance evaluation can timely grasp the operating status of the equipment. When the performance of the equipment drops to a certain threshold, even if the equipment can still work normally, to reduce losses or shorten downtime, the spare parts of the equipment should be prepared and replaced in time. Therefore, the first problem forces on how to carry out the performance evaluation and obtain accurate results.

**Problem 2:** The evaluation indexes selected for different equipment are also different. Generally, these indexes can reflect the equipment performance, but they are difficult to be used as the basis for spare parts management. The traditional spare parts management mode is determined according to the change law of equipment performance and expert knowledge. This approach is too subjective and unconvincing. The equipment needs to maintain good health in operation. Compared with equipment performance, The health degree of equipment reflects the ability or probability of equipment to perform its job successfully. Therefore, the second question focuses on how to obtain the health degree of equipment according to the performance envelope.

**Problem 3:** The purpose of spare parts management of large-scale equipment is to ensure that the equipment can work continuously, reduce losses, and shorten the downtime caused by equipment failures. Spare parts management mainly includes determining the number of spare parts and the accurate ordering time. The third problem is how to formulate a reasonable spare parts management strategy based on equipment health degree.

**Fig 1:**
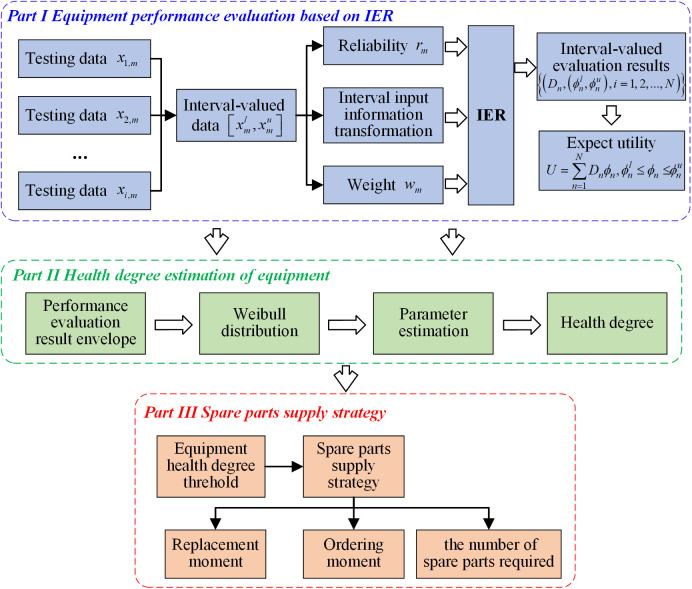
The detailed process of the proposed method.

These three problems correspond to the three modules of the proposed framework: IER-based performance evaluation, Weibull-based health degree estimation, and health-degree-driven spare parts ordering and replacement. The detailed process of the proposed method is shown in Fig 1.

## 3. Performance evaluation of large-scale equipment based on IER approach

The IER approach can effectively deal with uncertainty in the case of small samples and missing information. As the basis of spare parts management, it is expected to obtain the performance evaluation envelope of the same batch of a specific type of large-scale equipment. IER can be used to solve this problem.

Suppose that there are *M* samples. Due to the high cost of equipment, the number of test samples is limited, usually *M* ≤ 10. Select *L* indexes as performance evaluation indexes of the equipment. Collect testing data for evaluation indexes and the testing data $X_{v,l}^{m}$ can be obtained. m=1,2,…,M,l=1,2…,L,v=1,2,…,V. *V* is the number of testing times. Due to the small number of samples, there will be deviations if the statistical method is used to process the sample data. To retain the original information as much as possible, the sample data obtained at the same moment can be regarded as a group, and the maximum value and minimum value in the group of data can be respectively taken as the upper and lower bounds of the sample data interval at this moment.

Before using the IER approach for performance evaluation, different types of monitoring information should be uniformly transformed into belief structures. Therefore, an interval information transformation technique is used to deal with the interval information.

### Interval information transformation technique

There are various situations regarding the relative positions between interval values and attribute reference levels. The transformation of interval-valued inputs into belief structures can be divided into two cases: (1) the interval lies strictly between two adjacent referential values; (2) the interval spans across one or more referential grades. Depending on the specific case, the input information is transformed into a belief structure using an information transformation technique based on utility and rule-based reasoning. Let the interval-valued input be denoted as $\left[x_{i}^{-},x_{i}^{+}\right]$. After transformation, $\beta_{n,i}$ represents the belief degree assigned to the *n*-th referential grade of the *i*-th evidence, and *X*_*n*,*i*_ denotes the reference value of the *n*-th referential grade for the *i*-th evidence.

**Case 1: Interval values are within the two adjacent referential values.**

The belief degrees after transformation are as follows:


$$\begin{array}{c} \beta_{n,i}^{-}=\frac{X_{n+1,i}-x_{i}^{+}}{X_{n+1,i}-X_{n,i}},\beta_{n,i}^{+}=\frac{X_{n+1,i}-x_{i}^{-}}{X_{n+1,i}-X_{n,i}} \end{array}$$ (1)

$$\begin{array}{c} \beta_{n+1,i}^{-}=\frac{x_{i}^{-}-X_{n,i}}{X_{n+1,i}-X_{n,i}},\beta_{n+1,i}^{+}=\frac{x_{i}^{+}-X_{n,i}}{X_{n+1,i}-X_{n,i}} \end{array}$$ (2)

$$\begin{array}{c} \beta_{j,i}^{-}=\beta_{j,i}^{+}=0,j\neq n,n+1 \end{array}$$ (3)

Note that $\beta_{n,i}\in [\beta_{n,i}^{-},\;\beta_{n,i}^{+}]$, $\beta_{n+1,i}\in [\beta_{n+1,i}^{-},\;\beta_{n+1,i}^{+}]$. They are not independent and have to satisfy $\beta_{n,i}+\beta_{n+1,i}=1$.

**Case 2: Interval values span the referential grades.**

If the interval values cross a referential grade, the transformation of interval information is as follows:


$$\begin{array}{c} \beta_{n-1,i}^{-}=0,\beta_{n-1,i}^{+}=\frac{X_{n,i}-x_{i}^{-}}{X_{n,i}-X_{n-1,i}} \end{array}$$ (4)

$$\begin{array}{c} \beta_{n,i}^{-}=0,\beta_{n,i}^{+}=1 \end{array}$$ (5)

$$\begin{array}{c} \beta_{n+1,i}^{-}=0,\beta_{n+1,i}^{+}=\frac{x_{i}^{+}-X_{n,i}}{X_{n+1,i}-X_{n,i}} \end{array}$$ (6)

Note that $\beta_{n-1,i}\in \left[\beta_{n-1,i}^{-},\beta_{n-1,i}^{+}\right],\beta_{n,i}\in \left[\beta_{n,i}^{-},\beta_{n,i}^{+}\right],\beta_{n+1,i}\in \left[\beta_{n+1,i}^{-},\beta_{n+1,i}^{+}\right]$ and $\beta_{n+2,i}\in [\beta_{n+2,i}^{-},\beta_{n+2,i}^{+}]$. They are not independent and have to satisfy $\beta_{n-1,i}+\beta_{n,i}+\beta_{n+1,i}=1$.

**Remark 1:** if the interval values span several referential values, the information transformation process is the same with spanning one referential value. The belief degrees to the referential values, which are within the interval values, are in the range of $\left[\begin{array}{c} 0,1 \end{array}\right]$. The belief degree to the 1^*st*^ referential value is the same with (4), and the belief degree to the *n*-th referential value is the same with (6).

After information transformation, the interval-valued belief degrees can be obtained.

### Parameter calculation and evidence combination of IER approach

The parameters of the IER approach are the same as those of ER approach, including reliability and weight. The reliability of evidence reflects the ability of the evidence source to provide correct information, which is a statistical concept. The weight reflects the relative importance of evidence and has certain subjectivity. When the input information is interval value, the basic probability masses obtained are interval values regardless of whether the parameters are interval values.

Suppose that there are *L* pieces of evidence, and *N* referential grades. *H*_*n*_ is the evaluation grade, *e*_*i*_ represents the evidence, and meet the following condition:


$$\begin{array}{c} e_{i}=\left\{H_{n},\left(\beta_{n,j}^{-},\beta_{n,j}^{+}\right)\right\},i=1,\ldots ,L,n=1,\ldots ,N \end{array}$$ (7)

Extreme cases are considered here, and the parameters are assumed to be interval values. $w_{i}^{-}\leq w_{i}\leq w_{i}^{+}$, $r_{i}^{-}\leq r_{i}\leq r_{i}^{+}$. $w_{i}^{+}$ and $w_{i}^{-}$ $\left(0\leq w_{i}^{-}\leq w_{i}^{+}\leq 1\right)$ represent the upper and lower bounds of the interval of weight values respectively. $r_{i}^{+}$ and $r_{i}^{-}$ $\left(0\leq r_{i}^{-}\leq r_{i}^{+}\leq 1\right)$ represent the upper and lower bounds of the interval of reliability values respectively. $\tilde{w}$ is the hybrid weight represented by:


$$\begin{array}{c} {\tilde{w}}_{i}=w_{i}/\left(1+w_{i}-r_{i}\right) \end{array}$$ (8)

If *r*_*i*_ and *w*_*i*_ are interval values, the hybrid weight may be interval values, and the basic probability masses are given by:


$$\begin{array}{c} m_{n,i}=m_{i}(\begin{array}{c} H_{n} \end{array})\in [\begin{array}{c} m_{n,i}^{-},\;m_{n,i}^{+} \end{array}] \end{array}$$ (9)

$$\begin{array}{c} m_{H,i}=1-{\tilde{w}}_{i}\sum_{n=1}^{N}\beta_{n,i} \end{array}$$ (10)

$$\begin{array}{c} {\overset{―}{m}}_{H,i}={\overset{―}{m}}_{i}(\begin{array}{c} H \end{array})=(\begin{array}{c} 1-{\tilde{w}}_{i} \end{array})\in [\begin{array}{c} 1-{\tilde{w}}_{i}^{+},1-{\tilde{w}}_{i}^{-} \end{array}] \end{array}$$ (11)

$$\begin{array}{c} {\tilde{m}}_{H,i}={\tilde{m}}_{i}\left(H\right)\in \left[{\tilde{m}}_{H,i}^{-},{\tilde{m}}_{H,i}^{+}\right] \end{array}$$ (12)

where $\sum_{n=1}^{N}m_{n,i}+{\bar{m}}_{H,i}+{\tilde{m}}_{H,i}=1$. *m* is the basic probability mass. ${\tilde{w}}_{i}$ is the hybrid weight. ${\bar{m}}_{H,i}$ represents the basic probability masses generated by the relative importance of evidence and ${\tilde{m}}_{H,i}$ represents the basic probability masses caused by the incompleteness of the evaluation of evidence on the proposition. *m*_*H*,*i*_ is the residual probability mass after fusion. *n* = 1,...,*N*; *i* = 1,...,*L*.

Under interval constraint, the reasoning process of the IER approach becomes an optimization process. The combination of two basic probability masses *m*_1_ and *m*_2_ is as follows:


$$\begin{array}{c} m_{1-2}\left(H_{i}\right)=\frac{m_{1}\left(H_{i}\right)m_{2}\left(H_{i}\right)+m_{1}\left(H_{i}\right)m_{2}\left(H\right)+m_{1}\left(H\right)m_{2}\left(H_{i}\right)}{1-\sum_{k=1}^{2}\sum_{j=1,j\neq k}^{2}m_{1}\left(H_{k}\right)m_{2}\left(H_{j}\right)} \end{array}$$ (13)

$$\begin{array}{c} m_{1-2}(H)=\frac{m_{1}(H)m_{2}(H)}{1-\sum_{k=1}^{2}\sum_{j=1,j\neq k}^{2}m_{1}(H_{k})m_{2}(H_{j})} \end{array}$$ (14)

After the combination of all evidence, the belief degree of each evaluation grade can be obtained as follows:


$$\begin{array}{c} \beta_{n}=\frac{m_{n,e(L)}}{1-{\overset{―}{m}}_{H,e(L)}} \end{array}$$ (15)

$$\begin{array}{c} \beta_{H}=\frac{{\tilde{m}}_{n,e(L)}}{1-{\overset{―}{m}}_{H,e(L)}} \end{array}$$ (16)

where $\beta_{n}$ represents the belief degree to evaluation grade *H*_*n*_, and $\beta_{H}$ is the belief degree unassigned to any evaluation grades. n=1,2,⋯,N.

To facilitate the comparison or ranking of results, the expected utility method is used to transform the form of belief structure into the form of utility [45], as shown below:


$$\begin{array}{c} u(S(y_{i}))=\sum_{n=1}^{N}u(H_{n})\beta_{n}(y_{i}),\;i=1,\cdots ,L \end{array}$$ (17)

where *u*(*H*_*n*_) represents the referential value corresponding to the evaluation grade *H*_*n*_. $\beta_{n}(y_{i})$ represents the belief degree corresponding to the evaluation grade *H*_*n*_. *L* is the number of input indexes. *N* is the number of evaluation grades.

When multiple pieces of evidence exist, it should combine simultaneously. The optimization model proposed by Wang [46] is shown as follows:


$$\begin{array}{c} Max/min\;u=\sum_{n=1}^{N}u(H_{n})\beta_{n} \end{array}$$ (18)

$$\begin{array}{c} s.t.\;\beta_{n}=\frac{m_{n}}{1-{\overset{―}{m}}_{H}} \end{array}$$ (19)

$$\begin{array}{c} m_{n}=k[\prod_{i=1}^{L}(m_{n,i}+{\overset{―}{m}}_{H,i}+{\tilde{m}}_{H,i})-\prod_{i=1}^{L}({\bar{m}}_{H,i}+{\tilde{m}}_{H,i})] \end{array}$$ (20)

$$\begin{array}{c} {\tilde{m}}_{H}=k[\prod_{i=1}^{L}({\overset{―}{m}}_{H,i}+{\tilde{m}}_{H,i})-\prod_{i=1}^{L}{\overset{―}{m}}_{H,i}] \end{array}$$ (21)

$$\begin{array}{c} {\overset{―}{m}}_{H}=k[\prod_{i=1}^{L}{\overset{―}{m}}_{H,i}] \end{array}$$ (22)

$$\begin{array}{c} k={\left[\sum_{n=1}^{N}\prod_{i=1}^{L}\left(m_{n,i}+{\overset{―}{m}}_{H,i}+{\tilde{m}}_{H,i}\right)-\left(N-1\right)\prod_{i=1}^{L}\left({\overset{―}{m}}_{H,i}+{\tilde{m}}_{H,i}\right)\right]}^{-1} \end{array}$$ (23)

$$\begin{array}{c} m_{n,i}=m_{i}\left(H_{n}\right)={\tilde{w}}_{i}\beta_{n,i} \end{array}$$ (24)

$$\begin{array}{c} {\overset{―}{m}}_{H,i}={\overset{―}{m}}_{i}(H)=1-{\tilde{w}}_{i} \end{array}$$ (25)

$$\begin{array}{c} {\tilde{m}}_{H,i}={\tilde{m}}_{i}\left(H\right)={\tilde{w}}_{i}\beta_{H,i} \end{array}$$ (26)

$$\begin{array}{c} {\tilde{w}}_{i}=w_{i}/\left(1+w_{i}-r_{i}\right) \end{array}$$ (27)

$$\begin{array}{c} w_{i}^{-}\leq w_{i}\leq w_{i}^{+} \end{array}$$ (28)

$$\begin{array}{c} r_{i}^{-}\leq r_{i}\leq r_{i}^{+} \end{array}$$ (29)

where *i* is the number of evidence, *i* = 1,...,*L*. *n* is the number of referential grades, *n* = 1,...,*N*.

### IER evaluation model optimization method based on interval similarity

Since the evaluation model built above depends on expert experience and testing data, the interval values of performance evaluation results obtained using the above models are not optimal. The built evaluation model needs to be optimized to achieve the optimal performance evaluation envelope. Considering the actual values are interval values, a model optimization method based on interval similarity is presented.

Suppose that the actual performance of the equipment is interval value $p^{I}=[p^{l},p^{u}]$, and the performance evaluation result is interval value $y^{I}=[y^{l},y^{u}]$. $q_{j}(j=1,2,3,4)$ denotes the *j*-th largest of *p*^*l*^, *p*^*u*^, *y*^*l*^, *y*^*u*^. The similarity of the two interval values can be represented as follows:


$$\begin{array}{c} s\left(p,y\right)=\frac{\left(q_{2}-q_{3}\right)\left[1-sgn\left(y^{l}-p^{u}\right)\right]\left[1-sgn\left(p^{l}-y^{u}\right)\right]/4}{\left(q_{1}-q_{4}\right)-\left(q_{2}-q_{3}\right)\left|sgn\left(p^{l}-y^{u}\right)-sgn\left(y^{l}-p^{u}\right)\right|/2} \end{array}$$ (30)

where sgn(*x*) stands for SIGN function.

(30) shows that similarity reflects the degree of overlap between two interval values. Usually, 0≤s(p,y)≤1. The greater the overlap, the greater the similarity, the closer s(p,y) to 1. If two interval values overlap totally, s(p,y)=1; If two interval values overlap but do not exactly coincide, 0<s(p,y)<1; If two interval values do not overlap at all, s(p,y)=0.

The greater the similarity between the evaluation results and the actual values, the better the evaluation results are. Therefore, the following optimization objective function can be built.


$$\begin{array}{c} \left\{ \begin{array}{c} \max s\left(p,y\right)=\frac{\left(q_{2}-q_{3}\right)\left[1-sgn\left(y^{l}-p^{u}\right)\right]\left[1-sgn\left(p^{l}-y^{u}\right)\right]/4}{\left(q_{1}-q_{4}\right)-\left(q_{2}-q_{3}\right)\left|sgn\left(p^{l}-y^{u}\right)-sgn\left(y^{l}-p^{u}\right)\right|/2}\\ s.t.\;y^{I}=[y^{l},y^{u}],p^{I}=[p^{l},p^{u}] \end{array}\right. \end{array}$$ (31)

The interval similarity obtained in (31) is the actual and evaluation values. For the interval similarity under multiple test times, the optimization objective function should be modified as the sum of interval similarity under all times, which is represented as:


$$\begin{array}{c} \left\{ \begin{array}{cc} \max & \sum_{i=1}^{N}s_{i}\left(p,y\right)\\ s.t. & u=\sum_{n=1}^{N}u\left(H_{n}\right)\beta_{n}\\ & u^{I}=y^{I}=[y^{l},y^{u}]\\ & p^{I}=[p^{l},p^{u}] \end{array}\right. \end{array}$$ (32)

The performance evaluation envelope obtained by (32) may be more accurate than the initial performance evaluation envelope obtained by the initial optimization model.

The proposed model can be optimized using the fmincon function in MATLAB or other optimization methods. The evaluation results under different test times can be obtained by applying the above evaluation process to testing data at each moment once the envelope for this batch is also available.

## 4. Health degree estimation based on large-scale equipment performance

The above analysis can obtain the performance envelope of the large-scale equipment. Health degree of the equipment refers to the quantification level of the health and availability of the equipment, which is a way of probability expression of its workability. Therefore, the health degree analysis and evaluation of large-scale equipment is an important method to detect the health degree of equipment operation, and is also the basis of spare parts management.

The three-parameter Weibull distribution is developed based on the two-parameter distribution models. Its advantage lies in its adaptability to small sample sampling and experimental information. It has become a critical model in the statistical study of test samples’ reliability and health degree [47]. Since the normal distribution or Poisson distribution is too ideal, the Weibull distribution is relatively closer to reality. In particular, the Weibull distribution is more suitable for describing the performance degradation law of lifespan experiments [48]. The obtained performance degradation envelope (PDE) can be fitted with Weibull distribution, and the obtained PDE curve can be regarded as the equipment health envelope. Weibull distribution is used to fit the performance degradation envelope and estimate equipment health degree.

### Three parameter Weibull distribution

If random variable *z* follows the Weibull distribution with three parameters, which donates as Z~W(λ,k,μ), the PDE of the Weibull distribution is shown below:


$$\begin{array}{c} f\left(z,\lambda ,k,\mu \right)=\left\{ \begin{array}{cc} \frac{k}{\lambda }(\frac{z-\mu }{\lambda }{)}^{k-1}e^{-(\frac{z-\mu }{\lambda }{)}^{k}} & z\geq \mu \\ 0 & z<\mu \end{array}\right. \end{array}$$ (33)

where *z* is a random variable, λ is the scale parameter, *k* is the shape parameter, μ is the location parameter. If *k* = 1, the Weibull distribution becomes exponential distribution; If λ=1, The Weibull distribution becomes a minimized Weibull distribution.

The cumulative distribution function (CDF) of Weibull distribution is:


$$\begin{array}{c} F(z,\lambda ,k,\mu )=P(Z\leq z,\lambda ,k,\mu )=1-e^{-{\left(\frac{z-\mu }{\lambda }\right)}^{k}},z\geq \mu \end{array}$$ (34)

The mathematic expectation and variance of random variables which follow the three-parameter Weibull distribution are respectively as follows:


$$\begin{array}{c} E(\begin{array}{c} Z \end{array})=\mu +\lambda \Gamma (1+\frac{1}{k}) \end{array}$$ (35)

$$\begin{array}{c} Var\left(Z\right)=\lambda^{2}[\Gamma (1+\frac{2}{k})-\Gamma^{2}(1+\frac{1}{k})] \end{array}$$ (36)

where Γ(•) is the Gamma function:


$$\begin{array}{c} \Gamma (z)=\int_{0}^{\infty }\frac{t^{z}-1}{e^{t}}dt \end{array}$$ (37)

### Maximum likelihood estimation of three parameter Weibull distribution

The commonly used Weibull distribution parameter estimation methods are not accurate enough, and the amount of calculation is large, which will also be significantly affected by the initial value. Try to combine dichotomy with maximum likelihood estimation (MLE) to estimate parameters of Weibull distribution.

The basic idea of MLE is to select the undetermined parameter to maximize the probability of the sample appearing in the field of the observed value, and regard this most likely value as the estimated value of the parameter. For the PDE of Weibull distribution with three parameters, the likelihood function of *S* samples is:


$$\begin{array}{c} L\left(z_{1},z_{2},...,z_{S},\lambda ,k,\mu \right)=\prod_{s=1}^{S}f\left(z_{s},\lambda ,k,\mu \right)=\frac{k^{S}}{\lambda^{kS}}\prod_{s=1}^{S}{\left(z_{s}-\mu \right)}^{k-1}e^{-\sum_{s=1}^{S}{\left(\frac{z_{s}-\mu }{\lambda }\right)}^{k}} \end{array}$$ (38)

The MLE method aims to find the parameter value $\left(\begin{array}{c} \hat{\lambda },\hat{k},\hat{\mu } \end{array}\right)$ that maximizes the above equation. The usual way is by taking partial derivatives. The partial derivative of the above formula is too complicated, so the above formula can be converted to the following form.


$$\begin{array}{cc} \ln L(z_{1},z_{2},...,z_{s},\lambda ,k,\mu ) & =\sum_{s=1}^{s}\ln f\left(z_{s},\lambda ,k,\mu \right)\\ & =\sum_{s=1}^{s}\left(\ln\frac{k}{\lambda }+\left(k-1\right)\ln\frac{z_{s}-\mu }{\lambda }-{\left(\frac{z_{s}-\mu }{\lambda }\right)}^{k}\right)\\ & =\sum_{s=1}^{S}\ln\frac{k}{\lambda }+\left(k-1\right)\sum_{s=1}^{S}\ln\frac{z_{s}-\mu }{\lambda }-\sum_{s=1}^{S}{\left(\frac{z_{s}-\mu }{\lambda }\right)}^{k} \end{array}$$ (39)

Taking the logarithm does not change the maximum value of (39), and it also simplifies the derivation. When the likelihood function in (39) is a continuous differentiable function of the distribution parameters and the maximum value point is the inner point of the parameter interval, the maximum likelihood estimator of the distribution parameters needs to meet the following equations at the same time.


$$\begin{array}{cc} & \left\{ \begin{array}{c} \frac{\partial \ln L\left(z_{1},z_{2},\ldots ,z_{s},\lambda ,k,\mu \right)}{\partial \lambda }=\frac{k}{\lambda^{k+1}}\sum_{z=1}^{S}{\left(z_{z}-\mu \right)}^{k}-\frac{kS}{\lambda }=0\\ \frac{\partial \ln L\left(z_{1},z_{2},\ldots ,z_{s},\lambda ,k,\mu \right)}{\partial k}=\frac{S}{k}+\sum_{z=1}^{S}\ln\left(z_{z}-\mu \right)-S\ln\lambda -\sum_{z=1}^{S}\left[{\left(\frac{z_{z}-\mu }{\lambda }\right)}^{k}[\left(\ln\frac{z_{z}-\mu }{\lambda }\right)\right]=0\\ \frac{\partial \ln L\left(z_{1},z_{2},\ldots ,z_{s},\lambda ,k,\mu \right)}{\partial \mu }=(1-k)\sum_{z=1}^{S}\frac{1}{z_{z}-\mu }+\frac{k}{\lambda }\sum_{z=1}^{S}{\left(\frac{z_{z}-\mu }{\lambda }\right)}^{k-1}=0 \end{array}\right. \end{array}$$ (40)

In (40), *z*_*s*_ and μ can be represented by a new variable. Suppose that $\xi_{s}=z_{s}-\mu$, $\xi_{s}$ can be regarded as the input data. (40) can be revised as follows:


$$\begin{array}{c} \frac{k}{\lambda^{k+1}}\sum_{s=1}^{S}\xi_{s}^{k}-\frac{kS}{\lambda }=0 \end{array}$$ (41)

$$\begin{array}{c} \frac{S}{k}+\sum_{s=1}^{S}\ln\xi_{s}-S\ln\lambda -\sum_{s=1}^{S}\left[{\left(\frac{\xi_{s}}{\lambda }\right)}^{k}\left(\ln\frac{\xi_{s}}{\lambda }\right)\right]=0 \end{array}$$ (42)

$$\begin{array}{c} (\begin{array}{c} 1-k \end{array})\sum_{s=1}^{S}\frac{1}{\xi_{s}}+\frac{k}{\lambda }\sum_{s=1}^{S}(\frac{\xi_{s}}{\lambda }{)}^{k-1}=0 \end{array}$$ (43)

The following formula can be obtained by solving (41)


$$\begin{array}{c} \lambda =\sqrt[{k}]{\frac{\sum_{s=1}^{S}\xi_{s}^{k}}{S}} \end{array}$$ (44)

(44) represents the relationship between λ and *k*, λ=λ(k). Combine (43) and (44) to obtain the following equation:


$$\begin{array}{c} (\begin{array}{c} 1-k \end{array})\sum_{s=1}^{S}\frac{1}{\xi_{s}}+kS\sum_{s=1}^{S}\frac{\xi_{s}^{k-1}}{\sum_{s=1}^{S}\xi_{s}^{k}}=0 \end{array}$$ (45)

Then


$$\begin{array}{c} k=\frac{\sum_{s=1}^{S}\frac{1}{\xi_{s}}}{\sum_{s=1}^{S}\frac{1}{\xi_{s}}-S\sum_{s=1}^{S}\frac{\xi_{s}^{k-1}}{\sum_{s=1}^{S}\xi_{s}^{k}}} \end{array}$$ (46)

(46) represents the relationship between μ and *k*, μ=μ(k). Substituting μ=μ(k) and λ=λ(k) into (42), *f*_mle_(*k*) can be obtained:


$$\begin{array}{cc} f_{mle}(k) & =\frac{\partial \ln L}{\partial k}\\ & =\frac{S}{k}+\sum_{s=1}^{S}\ln\left(z_{s}-\mu (k)\right)-S\ln\lambda (k)\\ & \;-\sum_{s=1}^{S}\left[{\left(\frac{z_{s}-\mu (k)}{\lambda (k)}\right)}^{k}\left(\ln\frac{z_{s}-\mu (k)}{\lambda (k)}\right)\right] \end{array}$$ (47)

Compared with conventional numerical MLE algorithms, the proposed bisection-based MLE procedure does not require arbitrary initial guesses for the three Weibull parameters. By deriving λ=λ(k) and μ=μ(k), the estimation problem is transformed into finding the zero point of (47) within a physically meaningful interval of the shape parameter. Therefore, the convergence is mainly affected by the selected search interval rather than by subjective initial parameter guesses. For equipment health degree in engineering, the range of shape parameters *k* of Weibull distribution is usually in the interval range [1,10]. If the sign-change condition was satisfied, the bisection method guaranteed convergence, and the estimation error after *N* iterations was bounded by $(k_{u}-k_{l})/2^{N}$. If no zero point existed in the interval, a grid search was first used to locate the point that minimized (47), and the corresponding solution was treated as an approximate estimate.

### The detailed process of parameter estimation based on bisection method

The main idea of the bisection method is to divide the interval to be solved in half and find the interval where the zero point is located. The calculation is repeated until the zero point is found.

The detailed steps to find the zero point of (47) using the bisection method are as follows.

**STEP 1:** The range of shape parameter *k* is [1,10]. The upper bound of the range is *k*_1_ = 1, and the lower bound of the range is $k_{u}=10$. The values of Function *f*_mle_(*k*) at the boundary of the interval range are *f*_mle_(*k*_1_) and *f*_mle_(*k*_*u*_);

**STEP 2:** Calculate the value of *f*_mle_(*k*_*mid*_) at the middle point $k_{mid}=\frac{k_{1}+k_{u}}{2}$ of interval range $\left[\begin{array}{c} k_{1},k_{u} \end{array}\right]$. If $\left|f_{mle}\left(k_{mid}\right)\right|\leq \varepsilon$ (ε is the expected error), *k*_mid_ can be regarded the zero point of (47);

**STEP 3:** If $f_{mle}\left(k_{mid}\right)\cdot f\left(k_{u}\right)<0$, $k_{1}=k_{mid}$ and $k_{u}=k_{u};if\;f_{mle}\left(k_{mid}\right)f\left(k_{u}\right)>0$, $k_{u}=k_{mid}$ and $k_{1}=k_{1}$. Then go to **STEP 2** to continue the calculation.

This method does not require initial values and is simple and fast to iterate. At the same time, it will not be constrained by the number of samples. The bisection will be less computational, making it more suitable for engineering applications.

**Remark 2:** For the case where there is no zero in (47), you can try to find the *k*_min_ that minimizes. $\left|f_{mle}(k)\right|.k_{min}$ is used as the final solution, but *k*_min_ is not the best parameter estimate.

The CDF obtained by Weibull distribution can be regarded as the degenerate function of equipment. The function of health degree can be considered as follows:


$$\begin{array}{c} H\left(z,\lambda ,k,\mu \right)=1-P\left(Z\leq z,\lambda ,k,\mu \right)=e^{-{\left(\frac{z-\mu }{\lambda }\right)}^{k}},z\geq \mu \end{array}$$ (48)

It can be seen from (48) that the health degree function is a non-increasing function, which indicates that the health degree of equipment will decrease gradually as the working time.

## 5. Spare parts supply strategy based on large-scale equipment performance degradation

The basic idea of spare parts management is to find out the decline of equipment performance in time by evaluating the performance of essential components in the equipment, and replace the spare parts of the equipment in time before causing losses, to shorten the downtime and shutdown time of the equipment and ensure the excellent performance of the equipment [49].

For a complex system, when the performance of one equipment in the system decreases or fails, the whole system may be affected. If equipment with degraded or malfunctioning performance has available spare parts, the old equipment should be replaced with new equipment promptly. If there are no corresponding spare parts, the system must wait for available spare parts to be replaced. Due to the influence of spare parts production, transportation, and other factors, when there is a shortage of spare parts for equipment, the system may not work correctly, resulting in extended downtime. Therefore, the number of spare parts needs to be determined scientifically, and spare parts should be replaced and ordered in advance. The spare parts management process can be divided into two aspects: the number of spare parts and the timing of spare parts ordering and replacement.

### Determination of the number of spare parts based on multistate equipment health degree expectation

**Fig 2:**
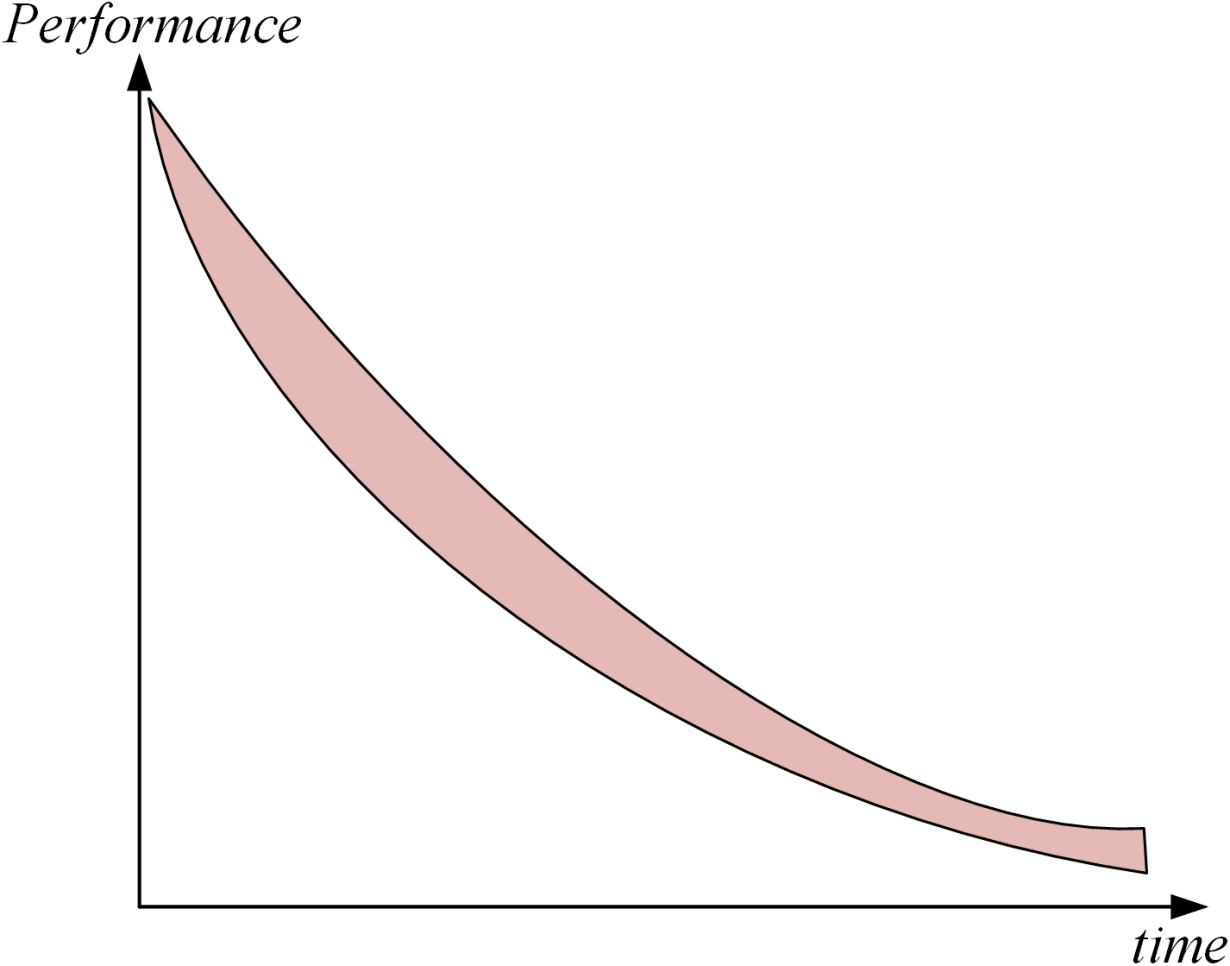
Performance evaluation envelope of equipment.

The number of spare parts needs to be determined according to the health degree of all currently used equipment. Since the influence of uncertain information and small samples are considered in the process of equipment performance evaluation, the obtained result is the equipment performance envelope, that is, the interval values evaluation results shown in Fig 2.

The shaded area in Fig 2 represents the performance evaluation envelope of a specific batch of equipment. The ordinate is the index reflecting the equipment performance, that is, the expected utility of the output index in Section 3. Assuming that the equipment is working, the performance evaluation result of the equipment is obtained according to the testing data of input indexes. Since the single measurement information is accurate, the precise value is taken as input information. The ER approach is used to solve the performance evaluation result under the condition of precise value input. The evaluation result should be interval value.

Suppose that at the moment *t*, *S* samples of the *b*-th equipment are obtained, denoted as $\left[z_{1}(t),z_{2}(t),\cdots ,z_{s}(t)\right]$. Through the content in Section 4, the parameter estimation value of the Weibull distribution can be calculated, and further the health degree assessment result can be obtained, denoted as $\left[R_{b}^{1}(t),R_{b}^{v}(t)\right]$.

Suppose that a total of *B* equipment is in use, and in engineering practice, the equipment is usually not of the same level of health degree. Health degree of the *b*-th equipment should be $\left[R_{b}^{1}(t),R_{b}^{v}(t)\right]$. To ensure that the equipment has enough spare parts for replacement in case of problems, it is necessary to prepare a corresponding number of spare parts in advance. The expected number of spare parts needed is calculated by using the expected health degree. Since the health degree $\left[R_{b}^{1}(t),R_{b}^{v}(t)\right]$ is interval value, the number of spare parts may also be interval-valued, and it should be:


$$\begin{array}{c} C_{1}(t)=\left\lfloor \sum_{b=1}^{B}\left(1-R_{b}^{u}(t)\right)\right\rfloor +1 \end{array}$$ (49)

$$\begin{array}{c} C_{𝔲}(t)=\lfloor \sum_{b=1}^{B}(1-R_{b}^{1}(t))\rfloor +1 \end{array}$$ (50)

where *C*_1_(*t*) and *C*_*u*_(*t*) are respectively the minimum and maximum number of spare parts required at the moment *t*. ⌊•⌋ is Floor function.

The interval value of the number of spare parts is [*C*_1_(*t*), *C*_*v*_(*t*)]. To ensure adequate spare parts for replacement in case of working equipment failure, the required number of spare parts should satisfy $C_{1}(t)=C_{v}(t)$.

### Spare parts ordering strategy considering time cost

The number of spare parts is changing dynamically. To meet the operational needs of the equipment, when the number of spare parts is insufficient, it is necessary to order and supplement in time. Due to the limitation of equipment production and transportation time, the replacement of spare parts will be delayed when the shortage of spare parts has occurred. Therefore, preventive replacement strategies should be developed.

#### 5.2.1. Analysis of remaining working time and additional number of spare parts.

**Fig 3:**
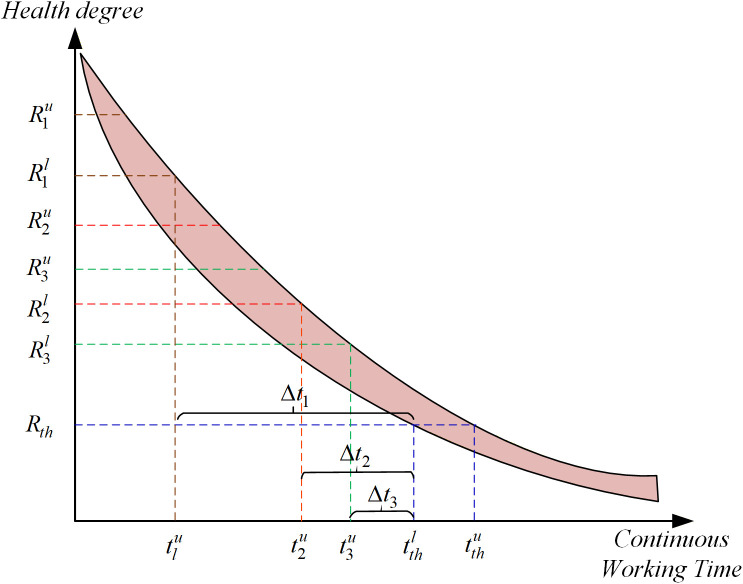
Health degree envelope of different equipment.

Suppose that a total of *B* equipment is in the working state. The working state of the equipment mentioned here is relative to the inventory status. In other words, the equipment is in use, not new or stored in a warehouse. The equipment is the same type and comes from the same manufacturer. Since each equipment has worked for a different length of time, the corresponding health degree may also be different. The equipment health degree interval values can be obtained with the method proposed in Section 4. Suppose that the $R_{b}^{v}$ and $R_{b}^{1}$ are respectively the upper and lower bounds of health degree of the b−th(b=1,2,...,B) equipment, shown in Fig 3. If the health degree of the *b*-th equipment is $\left[\begin{array}{c} R_{b}^{1},R_{b}^{v} \end{array}\right]$, to ensure that the number of spare parts obtained meets the needs, the minimum value ($i.e.\;R_{b}^{1}$) in the range $\left[\begin{array}{c} R_{b}^{1},R_{b}^{v} \end{array}\right]$ is taken as the health degree of the b-th equipment. Similarly, suppose that the working time interval corresponding to $R_{b}^{1}$ is $\left[\begin{array}{c} t_{b}^{1},t_{b}^{u} \end{array}\right]$, the maximum value in the range $\left[\begin{array}{c} t_{b}^{1},t_{b}^{u} \end{array}\right]$ is taken as its continuous working time.

Suppose *R*_th_ is the health degree threshold of equipment, when the health degree at this moment is lower than *R*_th_, the equipment needs to be replaced by spare parts. In Fig 3, $\Delta t_{b}=t_{th}^{1}-t_{b}^{v}$ can be regarded as the remaining time for the *b*-th equipment

At the initial moment *t*_0_, the required number of spare parts is $C_{t_{0}}$ using the method proposed in Section 5.1. The number of spare parts in stock is *C*_*store*_. If $C_{t_{0}}\leq C_{store}$, no additional spare parts are required; if $C_{t_{0}}>C_{store}$, it is necessary to replenish spare parts in time. At this time, additional number of spare parts should be:


$$\begin{array}{c} C_{\text{add}}=\left\{ \begin{array}{cc} 0, & if\;C_{t_{0}}\leq C_{\text{store}}\\ C_{t_{0}}-C_{\text{store}}, & if\;C_{t_{0}}>C_{\text{store}} \end{array}\right. \end{array}$$ (51)

where *C*_*add*_ represents the additional number of spare parts at the moment *t*_0_.

Since it also takes time to order and transport spare parts, it is necessary to order them in advance. In order to shorten the downtime of the equipment, a reasonable time for ordering spare parts should be determined.

#### 5.2.2. Spare parts preventive ordering strategy.

Before conducting the spare parts ordering timing study, make the following assumptions:

1. The health degree of equipment after replacement is [0.90, 0.95];
2. Regardless of the time it takes to replace the equipment, that is, spare parts can be replaced immediately when the equipment needs to be replaced;
3. The time required from placing an order for spare parts to transporting them into warehouses is $\Delta t_{tr}$, regardless of other spare parts ordering and transportation factors.

When $C_{add}=0$, it means that there is no need to order additional spare parts. When $C_{add}\neq 0$, it means that the quantity of spare parts to be ordered is *C*_add_, at this time, a spare parts ordering strategy needs to be formulated.

**Fig 4:**
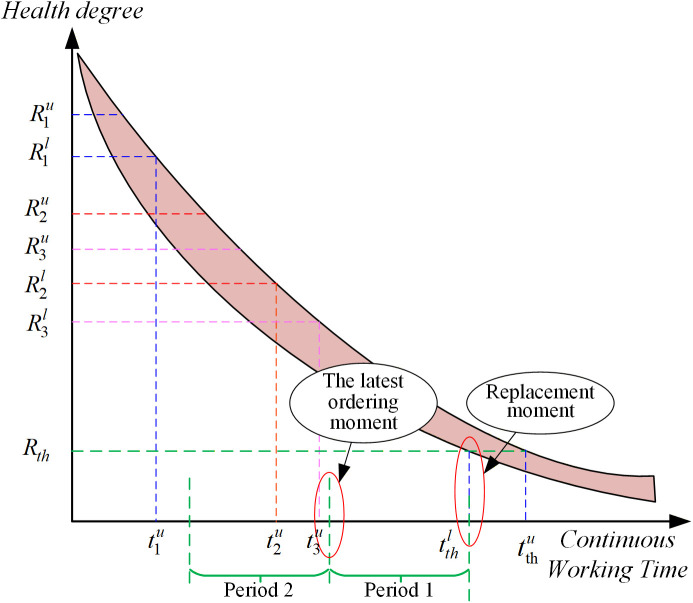
Order and replacement process of spare parts.

It is assumed that the time required for ordering and transporting spare parts is $\Delta t_{tr}$. The time $t_{th}^{1}$ corresponding to the health degree threshold *R*_th_ is taken as the replacement time of spare parts. Taking $t_{th}^{1}$ as the benchmark and $\Delta t_{tr}$ as the period, the time period $\left[t_{th}^{1}-\Delta t_{tr},t_{th}^{1}\right]$ is named time period 1, the time period $\left[t_{th}^{1}-2\Delta t_{tr},t_{th}^{1}-\Delta t_{tr}\right]$ is named time period 2, as shown in Fig 4. By analogy, the time period $\left[t_{th}^{1}-j\cdot \Delta t_{tr},t_{th}^{1}-(j-1)\cdot \Delta t_{tr}\right]$ is named period j. The discussion is carried out in the following 2 cases:

1. When $t_{0}\in [t_{th}^{1}-\Delta t_{tr},t_{th}^{1}]$, that is, the current time *t*_0_ is within time period 1, the spare par*t*s ordering demand should be issued immediately. Due to the limitation of transportation time, equipment downtime of length $\left(\begin{array}{c} \Delta t_{tr}-\Delta t_{i} \end{array}\right)$ may still be caused.
2. When $t_{0}\in [t_{th}^{1}-j\cdot \Delta t_{tr},t_{th}^{1}-(j-1)\cdot \Delta t_{tr}],j\geq 2$, that is, the current time *t*_0_ is within time period j(j⩾2), since the remaining working *t*ime is greater than the transportation time, there is no risk of equipment shutdown. At the same time. However, spare parts need to be stored for a period of time.

To comprehensively consider the operation requirements and storage costs of the equipment, on the premise of leaving a 10% margin, the spare parts ordering time is designed as follows:


$$\begin{array}{c} t_{or}=t_{th}^{1}-(1+10\%)\Delta t_{tr} \end{array}$$ (52)

Taking three pieces of equipment in use as an example, the order and replacement process of spare parts is shown in Fig 4. The 3^rd^, 2^nd^, and 1^st^ equipment will reach the ordering time one after another and order the corresponding number of spare parts.

The primary process of spare parts preventive ordering strategy considering time cost is as follows.

**STEP 1:** Determine the replacement moment $t_{th}^{l}$ of spare parts on the health degree envelope according to the health degree threshold *R*_th_;

**STEP 2:** Calculate the time required $\Delta t_{tr}$ to order and transport spare parts to the warehouse based on the actual transportation distance;

**STEP 3:** The initial moment is *t*_0_. If there is any working equipment during the period 1, it is necessary to analyze whether the spare parts in stock can meet the requirements when the equipment reaches $t_{th}^{l}$. If the spare parts in store cannot meet the requirements, the order of the spare parts needs to be started immediately to minimize equipment downtime;

**STEP 4:** If no equipment is in period 1, the number *B*(*t*_0_) of working equipment should be determined. Analyze and calculate the number of spare parts needed when all *B*(*t*_0_) pieces of equipment arrives in different working hours one by one, then the number of spare parts required can be calculated by (51);

**STEP 5:** When the equipment reaches the ordering time *t*_*or*_ in turn, spare parts orders should be issued according to the quantity;

**STEP 6:** When a certain piece of equipment has finished replacing spare parts, go to STEP 3 and continue with the above process.

**Fig 5:**
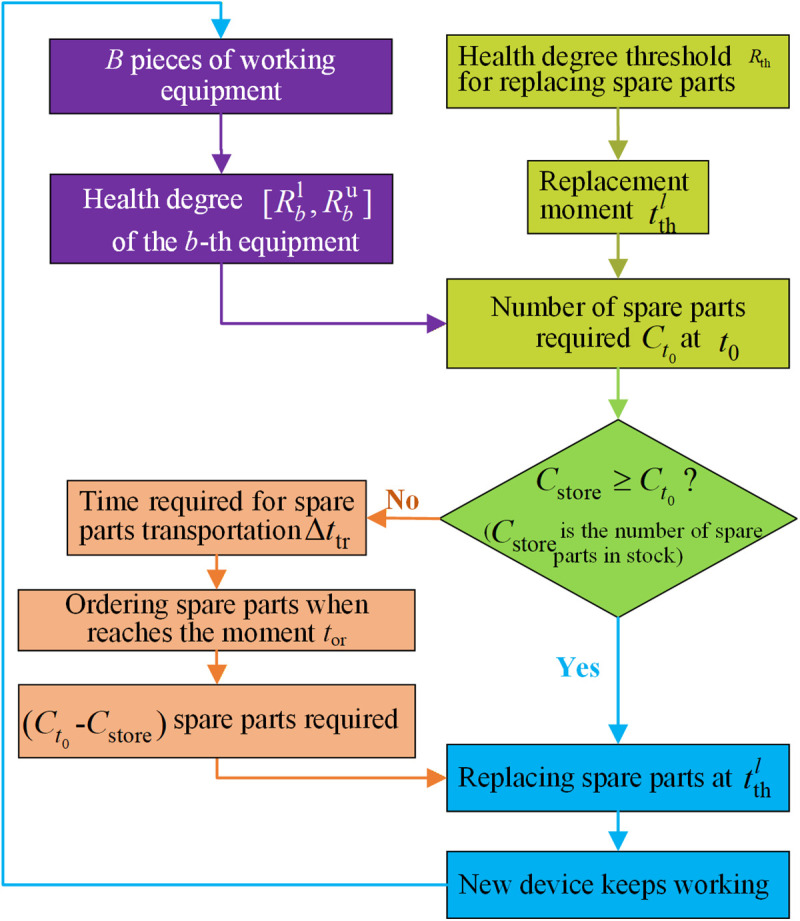
The detailed process of spare parts replacement moment determination.

The detailed process is shown in Fig 5.

Through the above two ways, the number of spare parts, the ordering and replacement time of spare parts can be determined to meet the health degree constraints.

## 6. Case study

Inertial Measurement Unit (IMU) is a system composed of various high-precision components such as gyros, accelerometers, servomotors, and rotary transformers. The system has a wide variety of parts and complex mechanisms, and can be considered as a type of large-scale and complex system. It has the advantages of comprehensive information, complete autonomy, highly concealed, free from time and geographical constraints, and is widely used in aerospace, aviation, navigation, and so on. As one of the core components of the inertial measurement unit, the gyroscope is used to sense the angle and angular velocity of the equipment relative to the inertial space. Its performance is critical to the measurement accuracy of the whole inertial unit. To show the validity of the proposed method, it is applied to the spare parts management of a specific type of laser gyroscope.

**Fig 6:**
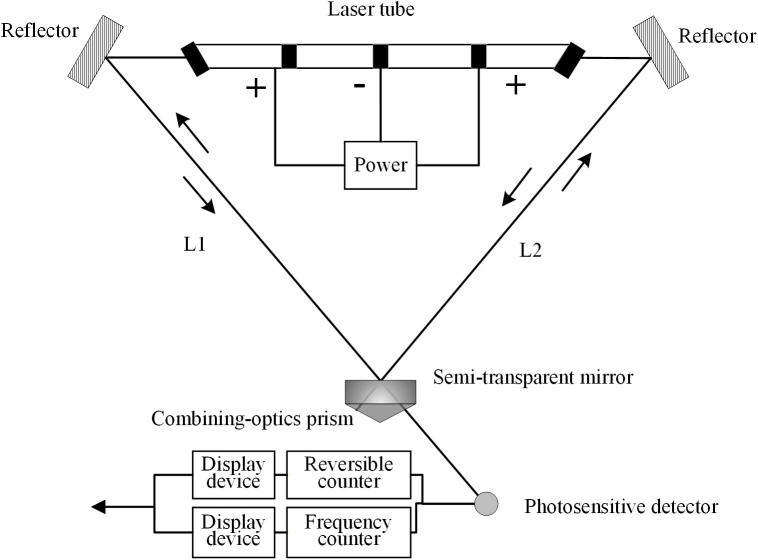
Laser gyro structure.

A laser gyroscope is an instrument that uses the Sagnac Effect to determine the physical orientation of an equipment’s movement accurately. Its structure is shown in Fig 6.

Compared with traditional mechanical gyroscopes, laser gyroscopes do not need the high-speed rotors, and have the characteristics of fast start-up, extensive dynamic range, high stability, strong anti-jamming ability, and extensive operating temperature range. Laser gyroscopes are ideal sensors for the strap-down inertial navigation system. As the core inertial equipment of aircraft inertial navigation system, laser gyroscope has been widely used in many fields such as satellite, submarine, navigation, and radar, and has become the primary basis for judging orientation in aviation, navigation, and space navigation systems.

### Construction of performance evaluation indexes system

In the long-term power-on test of laser gyroscope, there are a large number of monitoring indexes. Too many indexes make performance evaluation difficult. Suppose the selected index system contains too few indexes or omits the key ones which have a great impact on the performance of laser gyroscope. In that case, it is difficult to reflect the performance status of the laser gyroscope entirely and truly. It is easy to cause misjudgment. If too many indexes are selected, redundant indexes may be included to increase the complexity and workload of the evaluation process. Therefore, the first step in evaluating the performance of a laser gyroscope is to construct a good evaluation index system. At the same time, the selected index system should truly and comprehensively reflect the system’s performance and ensure the accuracy of the evaluation results.

The most significant factor affecting navigation accuracy is zero drift. The gyroscope error is a vital error source of the whole equipment control system, and the drift angular velocity is the main index to measure the accuracy of the gyroscope. Under the influence of the interference moment, the progress angular velocity of the gyro is called the drift angular velocity, which is reflected in the drift coefficient of the error coefficient in the drift error model of the gyro. The navigation accuracy obtained from the output information of the laser gyro is an important index to measure the gyro’s performance. Among many drift error coefficients, the most critical drift error coefficients are constant term $K_{0}$ and first-order term $K_{1}$. Therefore, the drift error coefficients $K_{0}$ and $K_{1}$ of the gyro are selected as input evaluation indexes and the static navigation error of the IMU as the output evaluation index. Five gyroscopes are chosen randomly from this batch of gyroscopes as evaluation objects. During the test process, the gyroscope works continuously. The zero term $K_{0}$ and first-order term $K_{1}$ of the gyroscope are tested and recorded with experimental facilities, and the navigation accuracy of the gyroscope is the output index.

**Fig 7:**
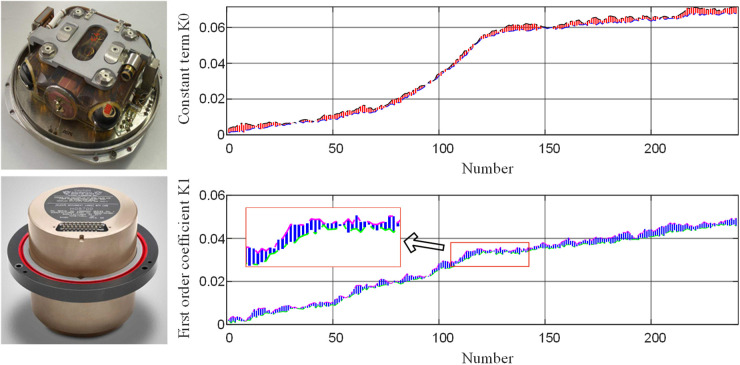
Trend of *K*_0_ and *K*_1_.

The testing data of the laser gyro used in this experiment was obtained by the manufacturer during the factory test. Five gyroscopes were selected from the batch as test samples. During 60 hours of continuous testing, 720 sets of testing data were obtained after testing every 5 minutes. Since there is too much testing data, a set of testing data is taken every 15 minutes, and 240 groups of data are obtained as input information. In Fig 7, $K_{0}$ and $K_{1}$ are both interval values. Laser gyroscope’s constant term and first-order coefficient increase with the working time. The size of $K_{0}$ and $K_{1}$ are proportional to the error of the output pulse of the gyroscope. Therefore, it can be seen from Fig 7 that the error of the gyroscope’s output pulse increases gradually, indicating that the gyroscope’s drift error increases gradually during continuous operation.

### Laser gyroscope performance evaluation based on IER approach

Evidence weight and reliability are two key parameters in an information retrieval system. Evidence weight reflects the relative importance of the evidence. Therefore, a coefficient of variation method based on the Monte Carlo approach is proposed to calculate the weights. The main idea of this method is to extract the interval-valued information of different indicators at each time point and then use the coefficient of variation method to compute the degree of dispersion of each indicator’s data. Different weight values can be obtained through multiple samplings, and the maximum and minimum values are used as the upper and lower bounds of the weight interval for each indicator. The theoretical basis for using the coefficient of variation method is that, in a comprehensive evaluation, an indicator with a larger degree of variation (i.e., higher data dispersion) carries more information and has a stronger ability to distinguish different states; therefore, it should be assigned a higher weight. The obtained weight ranges are then adjusted according to expert knowledge to ensure that the relative importance of different indicators aligns with expert judgment.

Reliability reflects the accuracy of the evidence provided by the evidence source. Accordingly, a statistical method based on Monte Carlo simulation is used to calculate the reliability of the evidence. This statistical method samples data from the interval values at each testing moment and determines whether the sampled value falls within the fluctuation interval determined by domain experts. The ratio of reliable counts to total counts in a single sample is calculated. A set of reliability values is obtained through multiple samplings, and the maximum and minimum values of this set are taken as the reliability interval. However, the setting of the aforementioned fluctuation interval involves a certain degree of subjectivity. To minimize subjective bias as much as possible, the method adopted in this paper is to determine the thresholds through the scoring of multiple experts. This approach transforms individual subjectivity into collective consensus to a certain extent, thereby enhancing the rationality and acceptability of the threshold settings.

The weights and reliability of the evidence are calculated using the above methods, and the corresponding results are obtained. The reliability calculation results are presented in Table 1.

**Table 1:** Value of reliability.

Index	Reliability
Constant term *K*_0_	[0.721, 0.800]
First-order coefficient *K*_1_	[0.817, 0.867]

**Fig 8:**
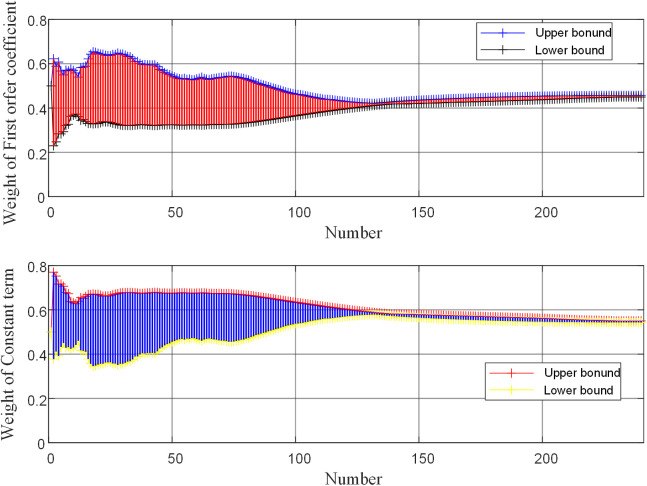
Weight of input indexes.

The result of the weight calculation is shown in Fig 8.

The red and blue areas in Fig 8 are ranges of $K_{0}$ and $K_{1}$, respectively. It can be seen that the weight range of $K_{0}$ is larger than that of $K_{1}$ in most cases, indicating that the coefficient of the constant term in the gyro error model is more important. This conclusion is also consistent with the engineering practice.

To put all input information into a unified evaluation framework for information fusion, all monitoring information needs to be transformed into the form of belief structure. According to the domain experts’ knowledge, set the referential grades of input information as (GOOD, AVERAGE, POOR) three different referential grades. The referential grades and values determined are shown in Table 2.

**Table 2:** Referential grades and values.

Index	Referential grades	Referential values
Constant		
term *K*_0_	(Good, Average, Poor)	(0, 0.041, 0.083)
First-order		
coefficient *K*_1_	(Good, Average, Poor)	(0, 0.025, 0.061)

**Fig 9:**
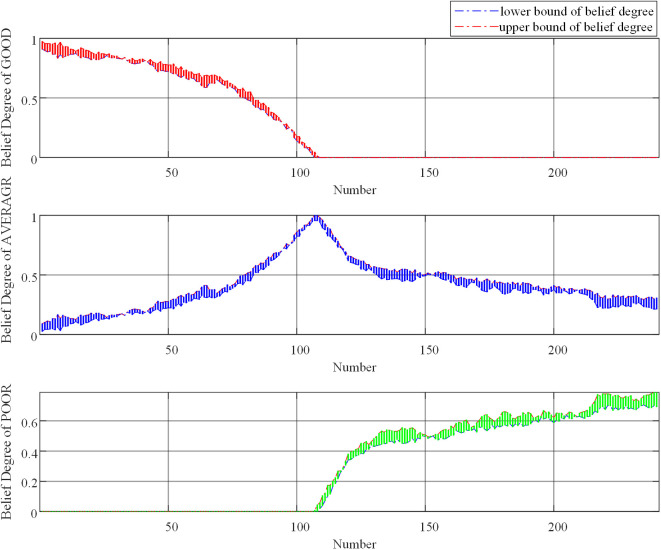
Belief structure of the constant term *K*_0_.

**Fig 10:**
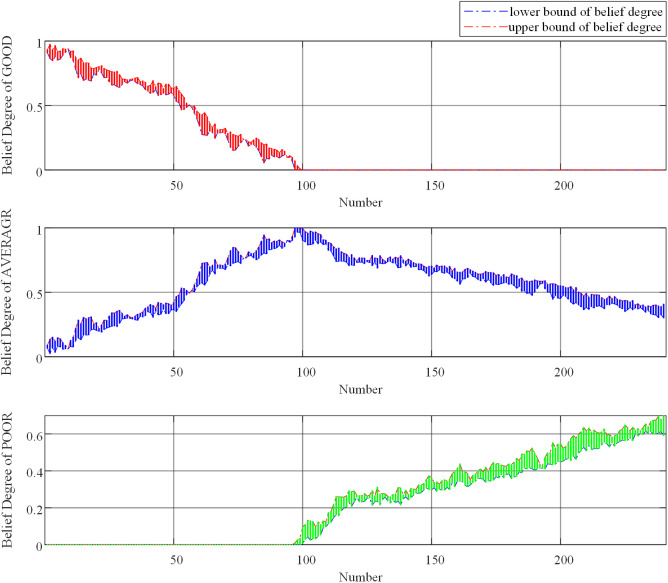
Belief structure of First order coefficient *K*_1_.

All monitoring information can be transformed into belief structure using the interval information transformation technique proposed in Section 3. The resulting interval belief structure is shown in Figs 9 and 10.

In Figs 9 and 10, the belief degrees for the red, blue, and green areas are GOOD, AVERAGE, and POOR, respectively. As the working time of the gyroscope increases, the belief degrees of GOOD decrease and the belief degrees of POOR increase, indicating that the gyroscope drift increases gradually.

**Fig 11:**
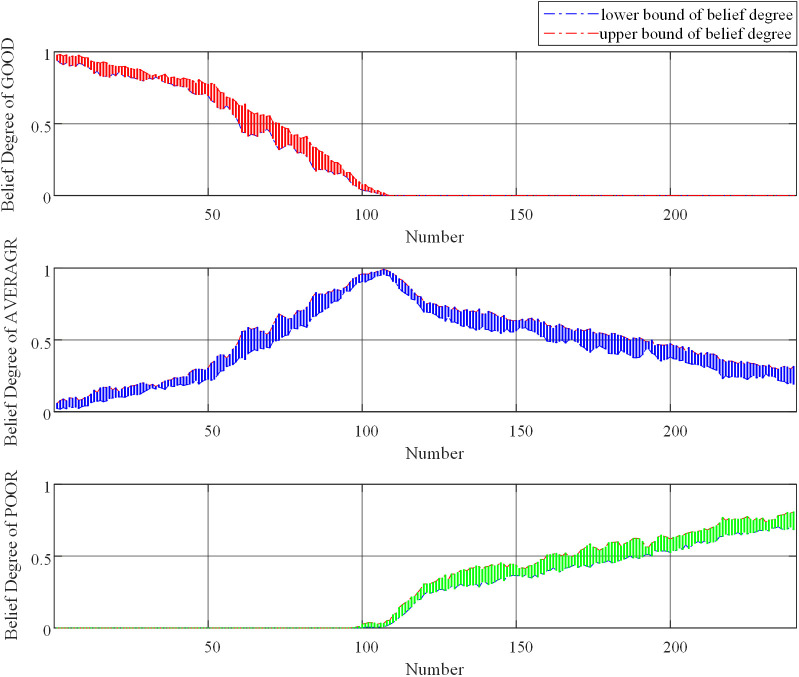
Belief structure of laser gyro evaluation results.

Based on the weight and reliability of the evidence, the belief structure is fused and evaluated using the IER approach. The optimization method based on interval similarity is used to get the optimal performance evaluation results of the laser gyroscope in the form of belief structure, as shown in Fig 11.

In Fig 11, the belief degrees of the laser gyroscope at different evaluation grades are obtained. During the early part of the test process, the belief degrees of GOOD decrease gradually, then become zero. The belief degrees of intermediate increase gradually in the early stage and decrease gradually in the later stage of the test. However, the belief degrees of POOR are zero in the early stage of the test process. As the test time increases, the belief degrees of POOR increase gradually. The above results show that the laser gyroscope’s performance decreases gradually during the test. The reason is that the drift coefficients of the gyroscope error model accumulate gradually with the test time, resulting in performance degradation.

**Fig 12:**
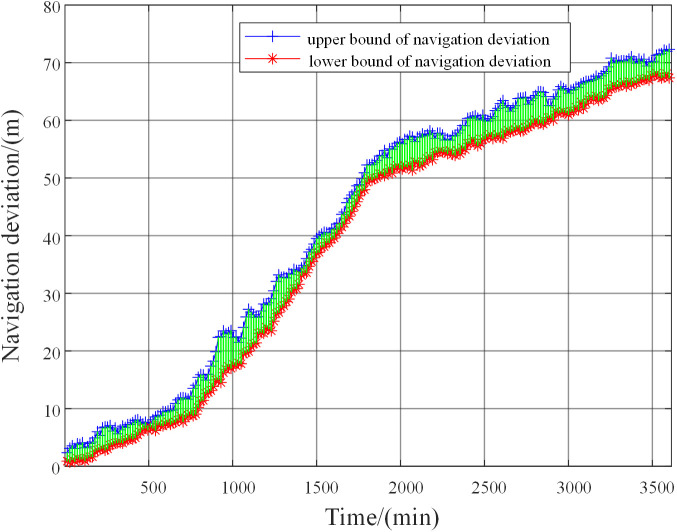
Expected utility of laser gyro evaluation results.

To visualize the degradation trend of performance evaluation results of laser gyroscopes, the evaluation results of the belief structure are transformed in the form of expected utility provided by (17). Referential values of the output index at different grades (0, 40, 80) are determined based on expert knowledge. The navigation deviation calculated for this type of laser gyroscope is shown in Fig 12.

In Fig 12, the utility values of navigation deviation increase gradually, reflecting that the laser gyroscope’s performance decreases gradually during continuous testing. The navigation deviation is calculated using the output pulses of the laser gyroscope increases more and more.

### Health degree estimation of laser gyroscope based on Weibull distribution

Weibull distribution is used to fit the performance degradation curve of this batch of laser gyroscopes, and the parameters of Weibull distribution are estimated by MLE based on the bisection method. Since the performance degradation data obtained is interval-valued, it is impossible to fit directly with the Weibull distribution. Interval-valued data refers to the that all data within that range may be possible. To make the Weibull distribution possible to contain all possible combinations of interval values, the Monte Carlo simulation method is used to sample all the interval values. Monte Carlo simulation can approximate the real parameter range gradually by repeated sampling. Fitting Weibull distribution parameters of laser gyroscope performance degradation envelope, 500 repeated sampling calculations were performed, and 500 groups of parameter estimates were obtained. The maximum and minimum values are taken as the upper and lower bounds of the Weibull distribution parameters, respectively. By sampling calculation, the parameter range of Weibull distribution corresponding to the performance degradation envelope is obtained, as shown in Table 3.

**Table 3:** Interval range of Weibull distribution parameters.

Parameters	Interval values
Scale parameter λ	[42.3417, 46.8058]
Shape parameter *k*	[1.4687, 1.7108]
Location parameter μ	[-0.2746, 0]

**Fig 13:**
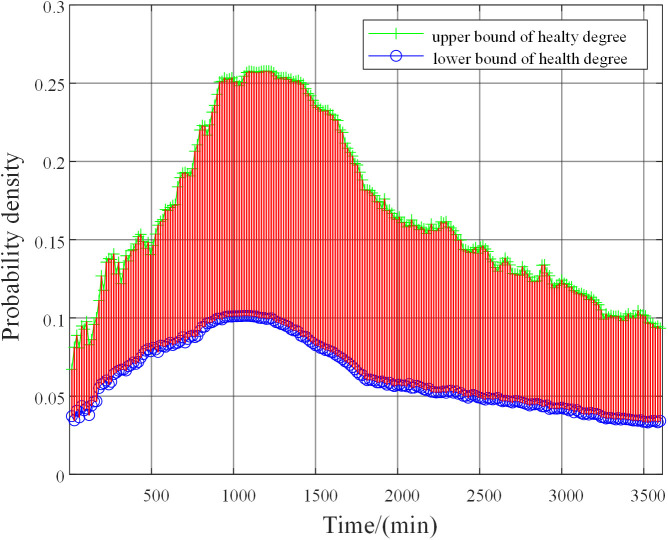
Probability density envelope.

According to the parameter values of Weibull distribution in Table 3, the upper and lower bounds of PDF can be obtained, as shown in Fig 13.

In Fig 13, the probability density envelope of the performance evaluation results of the laser gyroscope has a large range. This is because three parameters and input interval values constrain the Weibull distribution. The uncertainty of statistical calculation increases, and the probability density envelope obtained is also wider.

**Fig 14:**
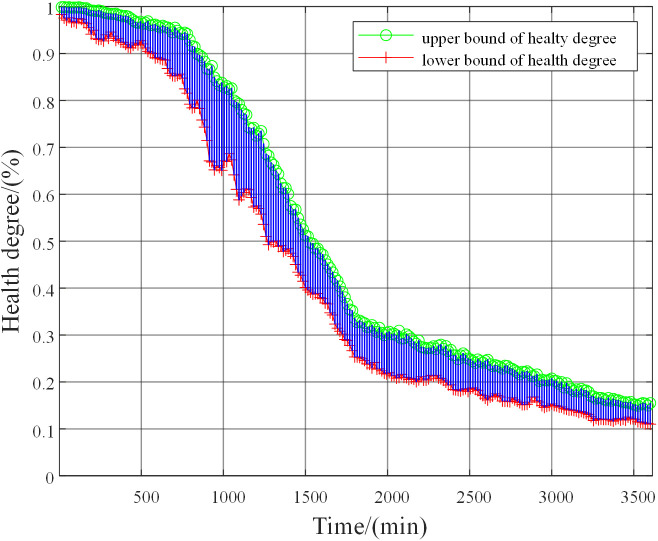
Health degree of laser gyro.

Based on the performance probability density envelope of the laser gyro, the health degree is estimated, and the health degree envelope is obtained, as shown in Fig 14.

In Fig 14, the shaded area represents the health degree envelope. The health degree envelope decreases with the increase of the gyroscope usage time, which reflects that the drift error of the laser gyroscope is accumulated gradually, resulting in a gradual decrease in its health degree, which is a non-increasing process.

Using the health degree of laser gyroscope as a basis can provide a basis for its spare parts management.

### Spare parts supply strategy for laser gyroscope considering performance degradation

The health degree of laser gyroscope is the basis for developing spare parts management. Spare parts management is also based on the performance status of the laser gyroscope at the current moment, to estimate the possible performance degradation of the laser gyroscope in the future.

Assume that there are four laser gyroscopes in use in this batch currently, and the health degree of the four laser gyroscopes are $H_{1}=[\begin{array}{c} 0.78,0.83 \end{array}],H_{2}=[\begin{array}{c} 0.64,0.73 \end{array}],H_{3}=[\begin{array}{c} 0.77,0.79 \end{array}],H_{4}=[\begin{array}{c} 0.45,0.55 \end{array}]$. According to (49) and (50), the required number of spare parts is $S=[\begin{array}{c} 2.55,2.86 \end{array}]$. To ensure spare parts are sufficient, the number of spare parts should be at least the minimum integer. That is, the number of spare parts required at the current moment is 3.

**Fig 15:**
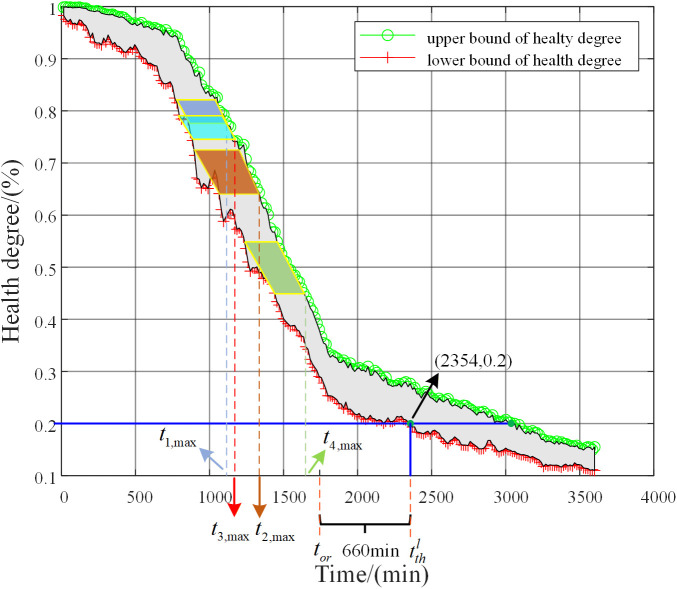
The process of spare parts supply strategy based on health degree.

Assume that based on domain expert experience, it is determined that when the health degree of the equipment reaches 0.2, replacement parts are required, and there are 3 spare parts stored in the warehouse currently. Based on the health degree function obtained in Section 4, the corresponding Minimum working time is 2354 min, that is $t_{th}^{1}=2354min$. Based on the health degree of four laser gyroscopes, the corresponding continuous working time interval range can be obtained, which are $t_{1}=[\begin{array}{c} 759,1109 \end{array}]$, $t_{2}=[\begin{array}{c} 891,1341 \end{array}],t_{3}=[\begin{array}{c} 798,1129 \end{array}],t_{4}=[\begin{array}{c} 1235,1637 \end{array}]$. Assume that the time from ordering to transporting the spare parts to the warehouse is $\Delta t_{tr}=600\min$. According to (52), the spare parts ordering time for each equipment is 660 minutes before $t_{th}^{1}$ (Fig 15).

However, the number of spare parts required is not the number of spare parts required at the current moment $\left(i.e.\;t_{0}\right)$. Instead, the number of spare parts needed at the time of replacement. Therefore, it is necessary to calculate the required number of spare parts at the moment that the 4^th^ laser gyroscope reaches $t_{th}^{1}$. The 4^th^ laser gyro reaches the critical value of health degree after 717 min, and needs to be replaced with spare parts. After 717 min, the lower bounds of the health degree range of the four laser gyroscopes are respectively $H_{1min}=0.2513,H_{2min}=0.2083,H_{3min}=0.2494,H_{4min}=0.2000$, as shown in Table 4.

**Table 4:** Health degree of 4 laser gyros at the moment (*t*_0_ + 717 min).

Number 1	*t*_0_	(*t*_0_ + 717 min)	Health degree
1	1109 min	1826 min	0.2513
2	1341 min	2058 min	0.2083
3	1129 min	1846 min	0.2494
4	1637 min	2354 min	0.2000

At this time, the number of spare parts required is $S_{min}=3.091$. Therefore, the number of spare parts required should be 4. Since only 3 spare parts are currently stocked, one more spare part needs to be ordered at $t_{0}+1013min$.

After replacing the spare parts of the 4^th^ laser gyro, its health degree is 0.95, and the number of spare parts in stock is reduced by 1. When the time reaches $t_{0}+508min$, the health degree of the 2^nd^ gyro reaches its replacement threshold, and should be replaced with spare parts. At this time, the status of each gyroscope is shown in Table 5.

**Table 5:** Health degree of four laser gyros at (*t*_0_ + 1013 min).

Number	Time worked at (*t*_0_ + 1013 min)	Health degree
1	2122 min	0.2071
2	2354 min	0.2000
3	2142 min	0.2069
4	453 min	0.9382

At this moment, the number of spare parts required is $S_{\min}=2.45$. Therefore, the number of spare parts required at the current moment is 3. After replacing the 4^th^ laser gyro, there are just 3 spare parts left. That means no new spare parts need to be ordered.

To sum up, until the 2^nd^ piece of equipment needs repair, a total of one spare part needs to be ordered at $t_{0}+57min$ to meet the operational needs of the equipment.

## 7. Conclusion

In engineering practice, the maintenance of large-scale equipment is costly and difficult. For such equipment, it is necessary to formulate a reasonable spare parts management strategy to ensure the number of spare parts and avoid waste. For the management of spare parts of irreparable equipment, this paper proposes a method to determine the number of spare parts required and the proper moment of ordering spare parts based on equipment performance. The IER approach is used to evaluate the performance of the same type of equipment, and the performance degradation envelope is obtained. The performance degradation envelope of the equipment is fitted by Weibull distribution, and the interval probability density function and the interval health degree are obtained. The health degree of the equipment is taken as the basis for replacing the spare parts, and the health degree threshold of the equipment needed to be replaced is determined according to expert knowledge. The number of spare parts required shall be determined based on health degree expectation. In addition, considering the ordering and delivery time of spare parts, the ordering time and quantity of spare parts are determined when multistate equipment work simultaneously using the health degree expectation. Finally, the proposed method is applied to a specific type of laser gyro, which provides theoretical support for the spare parts management of laser gyro.

The proposed method offers a feasible solution for the spare parts management of irreparable equipment and avoids relying solely on experience and knowledge for spare parts management. In future research, we need to focus on improving the accuracy of equipment performance evaluation and try to apply the proposed method to more practical equipment to continuously improve the method’s feasibility in engineering application.

## References

[1] Hekimoğlu M, Kök AG, Şahin M. Stockout risk estimation and expediting for repairable spare parts. Computers & Operations Research. 2022;138:105562. doi: 10.1016/j.cor.2021.105562

[2] Pham DT, Kiesmüller GP. Multiperiod integrated spare parts and tour planning for on-site maintenance activities with stochastic repair requests. Computers & Operations Research. 2022;148:105967. doi: 10.1016/j.cor.2022.105967

[3] Ding Y, Xia T, Wang Y, Zhang K, Li Y, Chen Z, et al. A multi-stage stochastic programming for joint condition-based replacement and spare parts ordering towards complex manufacturing system with uncertain lead times. Reliability Engineering & System Safety. 2025;264:111464. doi: 10.1016/j.ress.2025.111464

[4] Zhu M, Zhou X. Hypergraph-based joint optimization of spare part provision and maintenance scheduling for serial-parallel multi-station manufacturing systems. Reliability Engineering & System Safety. 2022;225:108619. doi: 10.1016/j.ress.2022.108619

[5] Rodrigues LR, Gomes JPP. Spare Parts List Recommendations for Multiple-Component Redundant Systems Using a Modified Pareto Ant Colony Optimization Approach. IEEE Trans Ind Inf. 2018;14(3):1107–14. doi: 10.1109/tii.2017.2767627

[6] Zhang L, Deng Q, Miao B, Liu X, Shao H. Parallel service mode of production and inventory for spare part inventory optimization. Knowledge-Based Systems. 2022;241:108282. doi: 10.1016/j.knosys.2022.108282

[7] Tang X, Xiao H, Kou G, Xiang Y. Joint Optimization of Condition-Based Maintenance and Spare Parts Ordering for a Hidden Multi-State Deteriorating System. IEEE Trans Rel. 2025;74(2):2503–14. doi: 10.1109/tr.2024.3385297

[8] Liu Y-Y, Chang K-H, Chen Y-Y. Simultaneous predictive maintenance and inventory policy in a continuously monitoring system using simulation optimization. Computers & Operations Research. 2023;153:106146. doi: 10.1016/j.cor.2023.106146

[9] Rausch M, Liao H. Joint Production and Spare Part Inventory Control Strategy Driven by Condition Based Maintenance. IEEE Trans Rel. 2010;59(3):507–16. doi: 10.1109/tr.2010.2055917

[10] Yildirim M, Sun XA, Gebraeel NZ. Sensor-driven condition-based generator maintenance scheduling—part I: Maintenance problem. IEEE Transactions on Power Systems. 2016;31(6):4253–62. doi: 10.1109/tpwrs.2015.2506600

[11] Ritchken PH, Fuh D. Optimal Replacement Policies for Irreparable Warrantied Items. IEEE Trans Rel. 1986;35(5):621–3. doi: 10.1109/tr.1986.4335572

[12] Hsieh C-C. Replacement and standby redundancy policies in a deteriorating system with aging and random shocks. Computers & Operations Research. 2005;32(9):2297–308. doi: 10.1016/j.cor.2004.03.004

[13] Park M, Jung KM, Park DH. A Generalized Age Replacement Policy for Systems Under Renewing Repair-Replacement Warranty. IEEE Trans Rel. 2016;65(2):604–12. doi: 10.1109/tr.2015.2500358

[14] Zhang Q, Xu P, Fang Z. Optimal age replacement policies for parallel systems with mission durations. Computers & Industrial Engineering. 2022;169:108172. doi: 10.1016/j.cie.2022.108172

[15] Beichelt F. A Generalized Block-Replacement Policy. IEEE Transactions on Reliability. 1981;R-30(2):171–2. doi: 10.1109/tr.1981.5221021

[16] Ma X, Han R, Chen Y, Qiu Q, Yan R, Yang L. Intelligent spare ordering and replacement optimisation leveraging adaptive prediction information. Reliability Engineering & System Safety. 2024;252:110420. doi: 10.1016/j.ress.2024.110420

[17] Wang Y, Zhu S, Li X, Liu Y. A Finite-Horizon Condition-Based Maintenance and Spare Parts Ordering Policy for a Two-Unit System Subject to Stochastic Dependence. Quality and Reliability Engineering International. 2026;42(2):749–66. doi: 10.1002/qre.70100

[18] Bai J, Pham H. Repair-Limit Risk-Free Warranty Policies With Imperfect Repair. IEEE Trans Syst, Man, Cybern A. 2005;35(6):765–72. doi: 10.1109/tsmca.2005.851343

[19] Wang H. A survey of maintenance policies of deteriorating systems. European Journal of Operational Research. 2002;139(3):469–89. doi: 10.1016/s0377-2217(01)00197-7

[20] Zheng M, Lin J, Xia T, Liu Y, Pan E. Joint condition-based maintenance and spare provisioning policy for a K-out-of-N system with failures during inspection intervals. European Journal of Operational Research. 2023;308(3):1220–32. doi: 10.1016/j.ejor.2023.01.011

[21] Ruiz-Castro JE. Preventive Maintenance of a Multi-State Device Subject to Internal Failure and Damage Due to External Shocks. IEEE Trans Rel. 2014;63(2):646–60. doi: 10.1109/tr.2014.2315922

[22] Lian J, Ren H, Sun Y, Hammerstrom DJ. Performance Evaluation for Transactive Energy Systems Using Double-Auction Market. IEEE Trans Power Syst. 2019;34(5):4128–37. doi: 10.1109/tpwrs.2018.2875919

[23] Alotaibi NM, Cavalcante CAV, Lopes RS, Scarf PA. Preventive replacement with defaulting. IMA Journal of Management Mathematics. 2020;31(4):491–504. doi: 10.1093/imaman/dpaa002

[24] Yang A, Qiu Q, Zhu M, Cui L, Chen W, Chen J. Condition-based maintenance strategy for redundant systems with arbitrary structures using improved reinforcement learning. Reliability Engineering & System Safety. 2022;225:108643. doi: 10.1016/j.ress.2022.108643

[25] Wang J, Luo L, Jin Y, Yang L. Joint optimization of maintenance policy and two-way stock transshipments policy for balanced systems. Reliability Engineering & System Safety. 2025;264:111345. doi: 10.1016/j.ress.2025.111345

[26] Wang J, Luo L, Mu G, Ma Y, Ni C. Joint optimization of quality control and maintenance policy for a production system with quality-dependent failures. Expert Systems with Applications. 2025;272:126800. doi: 10.1016/j.eswa.2025.126800

[27] Zhang Y, Cui R, Lv M, Li C, Deng Q. Predicting Performance Degradation on Adaptive Cache Replacement Policy. In: 2020 IEEE 26th International Conference on Parallel and Distributed Systems (ICPADS), 2020. 109–16. 10.1109/icpads51040.2020.00024

[28] Fang Z, Zhang Q, Cai J, Liu S. Optimal age replacement policies with multiple missions for multi-state systems. Computers & Industrial Engineering. 2022;163:107777. doi: 10.1016/j.cie.2021.107777

[29] Shen Y, Wang T, Song Z. Online performance and proactive maintenance assessment of data driven prediction models. J Intell Manuf. 2024;35(8):3959–93. doi: 10.1007/s10845-024-02357-8

[30] Yin X, He W, Wang J, Peng S, Cao Y, Zhang B. Health state assessment based on the Parallel–Serial Belief Rule Base for industrial robot systems. Engineering Applications of Artificial Intelligence. 2025;142:109856. doi: 10.1016/j.engappai.2024.109856

[31] Apeiranthitis S, Zacharia P, Chatzopoulos A, Papoutsidakis M. Predictive Maintenance of Machinery with Rotating Parts Using Convolutional Neural Networks. Electronics. 2024;13(2):460. doi: 10.3390/electronics13020460

[32] Fan H, Wu M, Cao W, Lai X, Chen L, Lu C, et al. An operating performance assessment strategy with multiple modes based on least squares support vector machines for drilling process. Computers & Industrial Engineering. 2021;159:107492. doi: 10.1016/j.cie.2021.107492

[33] Gao J, Lin T, He X, Bai J, Yuan L. Fuzzy comprehensive evaluation of mini pumped hydro storage based on combined weighting method. Journal of Energy Storage. 2026;152:120734. doi: 10.1016/j.est.2026.120734

[34] Wang Y, Li X, Yang C, Yang T, Lan F, Tian X. A dynamic Bayesian network risk assessment model for coal-fired power plants based on grey correlation and triangular fuzzy theory. Computer Standards & Interfaces. 2025;94:104001. doi: 10.1016/j.csi.2025.104001

[35] Jian-Bo Yang, Singh MG. An evidential reasoning approach for multiple-attribute decision making with uncertainty. IEEE Trans Syst, Man, Cybern. 1994;24(1):1–18. doi: 10.1109/21.259681

[36] Dempster AP. A Generalization of Bayesian Inference. Journal of the Royal Statistical Society Series B: Statistical Methodology. 1968;30(2):205–32. doi: 10.1111/j.2517-6161.1968.tb00722.x

[37] Yang J-B, Xu D-L. Evidential reasoning rule for evidence combination. Artificial Intelligence. 2013;205:1–29. doi: 10.1016/j.artint.2013.09.003

[38] Xu D-L, Yang J-B, Wang Y-M. The evidential reasoning approach for multi-attribute decision analysis under interval uncertainty. European Journal of Operational Research. 2006;174(3):1914–43. doi: 10.1016/j.ejor.2005.02.064

[39] Wang Y-M, Yang J-B, Xu D-L, Chin K-S. The evidential reasoning approach for multiple attribute decision analysis using interval belief degrees. European Journal of Operational Research. 2006;175(1):35–66. doi: 10.1016/j.ejor.2005.03.034

[40] Min Guo, Jian-Bo Yang, Kwai-Sang Chin, Hong-Wei Wang, Xin-Bao Liu. Evidential Reasoning Approach for Multiattribute Decision Analysis Under Both Fuzzy and Interval Uncertainty. IEEE Trans Fuzzy Syst. 2009;17(3):683–97. doi: 10.1109/tfuzz.2008.928599

[41] Feng Z, He W, Zhou Z, Ban X, Hu C, Han X. A New Safety Assessment Method Based on Belief Rule Base With Attribute Reliability. IEEE/CAA J Autom Sinica. 2021;8(11):1774–85. doi: 10.1109/jas.2020.1003399

[42] Sarkar A, Nlebedim IC, Shrotriya P. Performance degradation due to anodic failure mechanisms in lithium-ion batteries. Journal of Power Sources. 2021;502:229145. doi: 10.1016/j.jpowsour.2020.229145

[43] Wang H, Liao H, Ma X. Stochastic Multi-phase Modeling and Health Assessment for Systems Based on Degradation Branching Processes. Reliability Engineering & System Safety. 2022;222:108412. doi: 10.1016/j.ress.2022.108412

[44] Li XJ, Bin GF, Dhillon BS. Model to evaluate the state of mechanical equipment based on health value. Mechanism and Machine Theory. 2011;46(3):305–11. doi: 10.1016/j.mechmachtheory.2010.11.008

[45] Yang J-B. Rule and utility based evidential reasoning approach for multiattribute decision analysis under uncertainties. European Journal of Operational Research. 2001;131(1):31–61. doi: 10.1016/s0377-2217(99)00441-5

[46] Wang Y, Yang J, Xu D, Chin K. On the combination and normalization of interval-valued belief structures☆. Information Sciences. 2007;177(5):1230–47. doi: 10.1016/j.ins.2006.07.025

[47] Dong M, Nassif AB. Combining Modified Weibull Distribution Models for Power System Reliability Forecast. IEEE Trans Power Syst. 2019;34(2):1610–9. doi: 10.1109/tpwrs.2018.2877743

[48] Iqbal A, Jain T. Real-Time Event Detection Based on Weibull Distribution Using Synchrophasor Measurements for Enhanced Situational Awareness. IEEE Trans Power Syst. 2022;37(2):1425–36. doi: 10.1109/tpwrs.2021.3108481

[49] Tadikamalla PR. Age Replacement Policies for Weibull Failure Times. IEEE Trans Rel. 1980;R-29(1):88–90. doi: 10.1109/tr.1980.5220728

